# Eliciting End-State Comfort Planning in Children With and Without Developmental Coordination Disorder Using a Hammer Task: A Pilot Study

**DOI:** 10.3389/fpsyg.2021.625577

**Published:** 2021-01-28

**Authors:** Hilde Krajenbrink, Jessica Mireille Lust, Bert Steenbergen

**Affiliations:** ^1^Behavioural Science Institute (BSI), Radboud University, Nijmegen, Netherlands; ^2^Centre for Disability and Development Research (CeDDR), School of Behavioural and Health Sciences, Australian Catholic University, Melbourne, VIC, Australia

**Keywords:** motor planning, end-state comfort, developmental coordination disorder, children, task constraints

## Abstract

The end-state comfort (ESC) effect refers to the consistent tendency of healthy adults to end their movements in a comfortable end posture. In children with and without developmental coordination disorder (DCD), the results of studies focusing on ESC planning have been inconclusive, which is likely to be due to differences in task constraints. The present pilot study focused on the question whether children with and without DCD were able to change their planning strategy and were more likely to plan for ESC when demanded by complex object manipulations at the end of a task. To this end, we examined ESC planning in 18 children with and without DCD (aged 5–11years) using the previously used sword-task and the newly developed hammer-task. In the sword-task, children had to insert a sword in a wooden block, which could be relatively easily completed with an uncomfortable end-posture. In the hammer-task, children had to strike down a nail in a wooden pounding bench, which required additional force and speed demands, making it relatively difficult to complete the movement with an uncomfortable end-posture. In line with our hypothesis, the results demonstrated that children with and without DCD were more likely to plan for ESC on the hammer-task compared with the sword-task. Thus, while children with and without DCD show inconsistent ESC planning on many previously used tasks, the present pilot study shows that many of them are able to take into account the end-state of their movements if demanded by task constraints.

## Introduction

When selecting a grip in order to perform a grasping movement, several strategies can be used. Healthy adults show a consistent tendency to end movements in a comfortable posture, even if this comes at the expense of an uncomfortable start-posture, which is called the *end-state comfort (ESC) effect* (Rosenbaum et al., 1990). The results of studies in children are, however, inconclusive with regard to the onset of this ESC effect during development evidenced by varying percentages of ESC planning across varying age groups (Wunsch et al., 2013, for a review). A group of children in which ESC planning appears to be comprised, are those with a developmental coordination disorder (DCD; Adams et al., 2014, for a review). While the majority of studies found that children with DCD are less likely to plan for ESC compared to typically developing (TD) children (e.g., van Swieten et al., 2010; Wilmut and Byrne, 2014a; Fuelscher et al., 2016; Adams et al., 2017), mixed results regarding the differences between children with DCD and TD children are also reported (e.g., Smyth and Mason, 1997; Noten et al., 2014). These equivocal results, both among TD children and between TD children and children with DCD, seem to be due to differences in task constraints that are evident in the different studies (Jongbloed-Pereboom et al., 2016; Bhoyroo et al., 2019). This has led to the discussion as to whether optimizing ESC is the preferred strategy for children on all tasks (e.g., Wilmut and Byrne, 2014b; Krajenbrink et al., 2020). The role of task constraints has been recently highlighted in a multi-component account of motor skill performance and development in children with DCD (Blank et al., 2019). Central tenet of this account is the mutual interaction between individual, environmental, and task constraints that determines the resulting behavior. With regard to ESC planning, depending on the biomechanical costs of the start- and ensuing end-posture, children may use alternative strategies to achieve a task goal. Following this reasoning, children are expected to change their strategy to plan for ESC if demanded by complex object manipulations at the end of the task compared to simple object manipulations. In the present pilot study, we examined this expectation using two tasks that required a similar start-posture but differed with regard to the task demands.

One of the tasks on which performance of both TD children and children with DCD has been described as relatively poor with regard to ESC is the sword-task (Craje et al., 2010). In this task, children are asked to pick up a wooden sword and to subsequently stick it into a tight-fitting hole in a wooden block. For the so-called critical trials, the sword needs to be rotated first before the blade can be inserted into the wooden chest. Jongbloed-Pereboom et al. (2013) examined performance across age on the sword-task among 3–10years old TD children and found that the percentage of ESC on critical trials increased from about 20% for the youngest age group to about 60% for the oldest age group. When compared to the overturned cup task (i.e., turning an upside-down cup upright) and the bar transport task (i.e., placing a horizontal bar in a target standard), percentages of ESC on the sword-task were the lowest, both for TD children, adolescents, and even adults (Jongbloed-Pereboom et al., 2016). Adams et al. (2016) and Adams et al. (2017) assessed the sword-task among a group of 6–11years old TD children and children with DCD and found lower percentages of ESC on the critical trials in the DCD group compared with their TD peers. This decreased tendency to plan for ESC has been interpreted as either a deficit or a developmental delay in motor planning in children with DCD.

However, these relatively low ESC percentages on the sword-task can be understood if we take a closer look at the way the task is set up. The sword-task can be relatively easily completed with a comfortable start-posture that results in an uncomfortable end-posture. At the same time, however, the postural demands of the initial uncomfortable start-posture that is necessary in order to achieve ESC are relatively high (Jongbloed-Pereboom et al., 2016). Thus, based on the relative (dis)comfort of the start- and end-posture, children may as well use the easiest initial grip to complete the task goal. This could be particularly true for children with DCD as the costs related to a biomechanically uncomfortable start-posture may be higher for them due to their motor difficulties (Wilmut and Byrne, 2014a). In other words, striving for ESC may not always be the most efficient strategy. Indeed, studies that focused on varying strategies used by children to solve motor planning tasks, found that next to ESC planning, children with and without DCD also use planning strategies based on start-state comfort, minimal initial rotation, or repetition of the previous movement (Wilmut and Byrne, 2014b; Bhoyroo et al., 2018).

**Figure 1:**
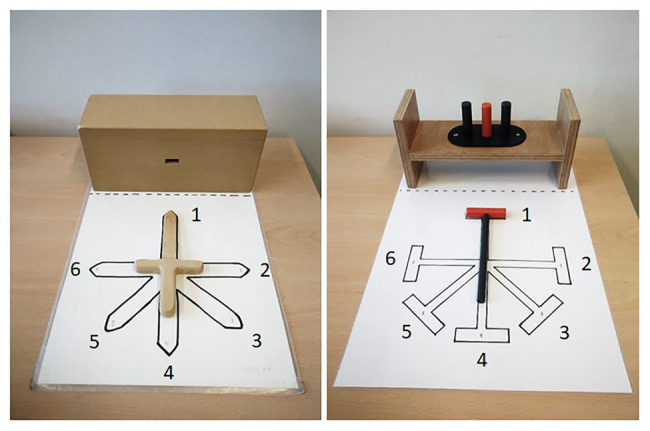
Sword-task (left) and hammer-task (right) with the sword/hammer in orientation 1.

It is, therefore, interesting to examine whether children may change their strategy to strive for ESC if the relative weight of the costs and benefits of an uncomfortable start- or end-posture change. It is assumed that by ending in a comfortable posture with the joints in a mid-range position, subsequent object manipulations can be performed with more precision (Short and Cauraugh, 1999). Thus, if the precision demands of the to-be-performed manipulation at the end of the task are higher, it is expected that it is more beneficial to end the movement in a comfortable posture in order to complete the required task goal. Following this reasoning, we developed a hammer-task in which children needed to pick up a hammer to strike down a nail in a wooden pounding bench (Figure 1). A hammering task has been used before to measure ESC in children (Comalli et al., 2016). The postural demands of the start of the movement are equal to the sword-task as well as the demanded end point precision. Importantly, however, the hammer-task incorporates additional complexity since sufficient force and speed needs to be exerted while hammering. This combination of precision, force, and speed demands for the hammer-task was hypothesized to lead to a higher degree of ESC planning compared with the sword-task, as a non-ESC planning strategy would result in poor precision and power when striking the nail. In addition, we hypothesized that this would be particularly true for children with DCD compared with TD children.

## Materials and Methods

### Participants

Participants were nine children (six boys and three girls) with DCD and nine gender and age-matched (within 8months, except for one child that was matched within 14months) controls that also participated in a larger study on motor planning as reported in Krajenbrink et al., in prep. Children were 5–11years old (*M*=8y0m, *SD*=2y0m). The children with DCD met the following inclusion criteria based on the DSM-V criteria: a Movement Assessment Battery for Children 2 (Henderson et al., 2007) total score ≤16th percentile or component score ≤5th percentile (criterion A), treated or have been treated for a motor coordination problem by a pediatric physical therapist and interference of the motor difficulties with daily activities, measured using two parent questionnaires, namely the Developmental Coordination Disorder Questionnaire (DCD-Q, Dutch translation; Schoemaker et al., 2008) and the DCDDaily-Q (van der Linde et al., 2015; criterion B), early onset of symptoms (criterion C); and no report of any cognitive impairment, visual impairment, or neurological deficit that would explain the child’s motor difficulties (criterion D). Comorbid disorders were Attention Deficit Hyperactivity Disorder (2) and Attention Deficit Disorder (1) as reported by parents. All TD children had a mABC-2 score >16th percentile. In addition, parents completed an ADHD-questionnaire as a descriptive measure of ADHD symptoms (AVL; Scholte and van der Ploeg, 2004). Child characteristics including the questionnaire scores are presented in Table 1. The study was approved by the Ethics Committee of the Faculty of Social Sciences at Radboud University (ECSW-2019-122).

**Table 1:** Child characteristics for DCD group and TD group.

	DCD group	TD group
Age in years (*SD*)	8y,0m (2y,1m)	8y,0m (2y,0m)
Sex (male/female)	6/3	6/3
Dominant hand (left/right)	0/9	3/6
mABC-2 *M* (*SD*)	2.30 (2.93)	50.00 (19.92)
AVL *M* (*SD*)	28.83 (11.58)	13.17 (8.61)
DCD-Q *M* (*SD*)	28.56 (6.84)	64.11 (9.58)
DCDDaily-Q participation *M* (*SD*)	47.83 (7.28)	34.56 (3.64)
DCDDaily-Q activities *M* (*SD*)	52.22 (6.76)	31.89 (6.88)
DCDDaily-Q learning *M* (*SD*)	18.11 (3.26)	0.89 (1.69)

### Materials

The previously administered sword-task (Craje et al., 2010) and the newly developed hammer-task were used as a measure of second-order motor planning. Both tasks are depicted in Figure 1. In the sword-task (left picture), children were asked to pick up the sword and to subsequently stick it into the hole of the wooden block. In each trial, the experimenter placed the sword on the template board in one of the six sword orientations. In the hammer-task (right picture), children were asked to pick up the hammer and to subsequently strike down the middle nail in the wooden pounding bench. Here, each trial, the hammer was placed on a similar template board with six hammer orientations. The other two nails were added to increase precision demands. As can be seen in Figure 1, for both tasks, two orientations were critical orientations (i.e., orientations 2 and 3 for right-handed children and orientations 5 and 6 for left-handed children) for which children had to sacrifice comfort of their start grip in order to end the task in a comfortable position (i.e., critical trials). The other four orientations served as control orientations for which a comfortable start grip resulted in a comfortable end position (i.e., non-critical trials). For both tasks, each orientation was repeated three times in a pseudo-random order with all six rotations appearing every six trials, resulting in a total of 18 trials per task. A score of 1 (i.e., action ended in an ulnar deviation, with the thumb toward the blade/hammerhead) or 0 (i.e., action ended in a radial deviation, with the thumb away from the blade/hammerhead) was assigned for each trial for each child. The proportion comfortable end postures were the outcome measure.

### Procedure

The hammer-task was appended to the study procedure of a larger data collection reported in Krajenbrink et al., in prep. As part of this larger data collection, three second-order motor planning tasks were examined in counter-balanced order, one of which being the sword-task. For the final 9 children that were included in the DCD group and the final 29 children in the TD group, the hammer-task was added at the end of the protocol. The data from these children with DCD and a gender and age-matched selection of 9 TD children (children were selected randomly in case of multiple options) were included in the present study. Children were seated and could comfortably reach the experimental materials. Before examining the second-order motor planning tasks, hand preference was determined by asking children to write their name down on the session form. For most TD children, data collection took place at their school and for most children with DCD, data collection to place at their home. Completing the sword-task and the hammer-task took about 5min in total.

### Data Analysis

In order to examine whether performance differed between the sword-task and hammer-task for children with DCD and TD children, a generalized linear mixed-effects model with a binomial link function was performed using the glmer function of the lme4 package (Bates et al., 2015) in R (R Core Team, 2020). It was decided to use this analysis instead of a more traditional approach as it is most suitable for binomial data. In the model, performance (proportion of ESC on the critical orientations, included as the number of critical trials ending in ESC and the number of critical trials not ending in ESC) was predicted as a function of the fixed effect of group (DCD or TD), the fixed effect of task (sword or hammer), as well as the interaction thereof. A random intercept for participant was included in order to control for individual variances across measurements. The model ran without warnings and provided a good fit of the data. Model diagnostic plots (i.e., a distribution of the residuals and a plot of the residuals as a function of the fitted values) yielded no indication of violations of the assumptions of normality, homoscedasticity, and linearity. Finally, there were no standardized residuals with values below −2.0 or above 2.0. Values of *p* are based on confidence intervals that were calculated with the confint function using bootstrap resampling. The beta coefficients that resulted from the model were converted into odds ratios (ORs). It should be noted here that the results of this model must be interpreted with caution as the small sample size has inherent limitation with respect to the reliability of the estimates and the generalizability of the results.

## Results

**Figure 2:**
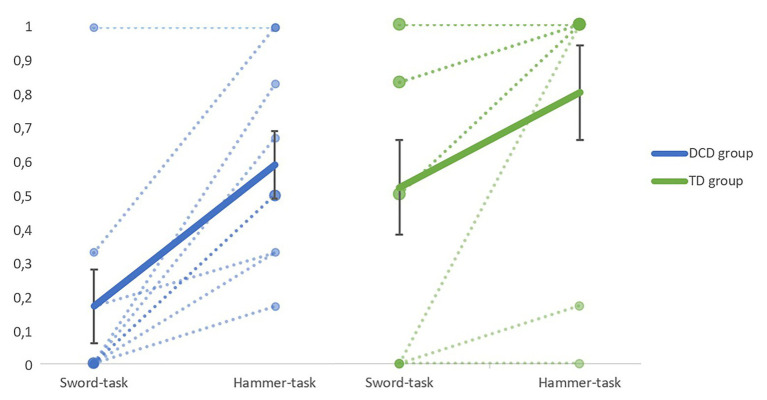
Proportion of end-state comfort (ESC) on the critical trials of the sword-task and hammer-task, developmental coordination disorder (DCD) group on the left, and typically developing (TD) group on the right. Dotted lines represent individual data, with the diameter of the end caps being proportional to the number of cases. The fat lines represent the group average and error bars represent SEs.

Descriptive statistics of both the critical and the non-critical trials of the sword-task and the hammer-task are represented in Table 2. The main variable of interest was the proportion of ESC on the critical trials of the sword-task and the hammer-task, which is represented in Figure 2 for children with DCD and TD children separately. Fourteen children performed better on the critical trials of the hammer-task compared with the sword-task. The other four children performed equal on both tasks, with three of them having the maximum score on both tasks. When looking at both groups separately, on the sword-task, six TD children ended half or more of the trials in ESC, but the other three TD children ended none of the trials in ESC. In the DCD group, only one child ended half or more of the trials in ESC and six children ended none of the trials in ESC. On the hammer-task, seven TD children ended all trials in ESC, but the other two children still ended less than half of the trials in ESC. Two children in the DCD group ended all of the trials in ESC and another four children ended half or more of the trials in ESC. Three children with DCD ended less than half of the trials in ESC.

**Table 2:** Proportion of trials ending in ESC for the critical and non-critical orientations for the DCD group and TD group.

		DCD group	TD group
Sword-task	Critical trials *M* (*SD*)	0.17 (0.33)	0.52 (0.43)
	Non-critical trials *M* (*SD*)	0.89 (0.10)	0.98 (0.04)
Hammer-task	Critical trials *M* (*SD*)	0.59 (0.30)	0.80 (0.41)
	Non-critical trials *M* (*SD*)	0.98 (0.04)	1.0 (0.00)

Results of the generalized linear mixed-effects model with a binomial link function showed a significant main effect of task, indicating that the average proportion of ESC was higher on the hammer-task compared to the sword-task, *OR*=0.02, *b*=−3.78, *SE*=0.88, *z*=−4.32, *p*<0.05, 95% CI [−6.42, −2.16]. The main effect of group, *OR*=14.76, *b*=2.69, *SE*=1.85, *z*=1.45, *p*>0.05, 95% CI [−0.48, 12.51], and the interaction between task and group, *OR*=0.75, *b*=−0.29, *SE*=1.51, *z*=−0.19, *p*>0.05, 95% CI [−7.82, 2.60], were not statistically significant. This indicates that the difference in performance on both tasks was not statistically different between children with DCD and TD children.

## Discussion

The aim of this pilot study was to examine whether children with and without DCD are more likely to plan for ESC when demanded by complex object manipulations at the end of the task. To this end, children with and without DCD performed the newly developed hammer-task after completing the previously used sword-task. In contrast to the sword-task, completing the hammer-task requires sufficient force and speed to hammer the nail down, making it more difficult to complete the goal of the task with an uncomfortable end-posture. We found that both children with DCD and TD children were more likely to strive for ESC on the hammer-task compared with the sword-task. Below, we will discuss these results in more detail.

In line with our expectation, we found that almost all children were more likely to sacrifice comfort of the start-posture and end the movement in a comfortable posture when completing the hammer-task as compared with the sword-task. Clearly, the additional force and speed demands in the hammer-task elicited more planning for ESC. This supports the multi-component account proposed by Blank et al. (2019) which stresses the role of task constraints, in interaction with environmental and individual constraints, to explain the behavior of children with DCD. In addition, previous studies on ESC planning also suggested that performance is task dependent (Knudsen et al., 2012; Jongbloed-Pereboom et al., 2016; Bhoyroo et al., 2019). In contrast to these previous studies, however, in our study, an increase in task demands was associated with increased percentages of ESC. In the hammer-task, additional force and speed demands led to a higher use of the ESC optimization strategy. Thus, it seems that the relative costs and benefits of an uncomfortable start- and end-posture determine what strategy children use. Although we assume that the benefits of ending in ESC are higher in the hammer-task compared with the sword-task, our paradigms did not provide an objective measure to support this claim. Future research is, therefore, warranted in which a measure that reflects the efficiency of task completion is included (e.g., accuracy, speed, or force) in order to test whether a comfortable end-posture from an adult perspective, is also beneficial for children with and without DCD.

Collectively, the results suggest that if children, both TD and children with DCD, fail to show a high percentage of ESC in a certain task, this does not necessarily mean that they are unable to take into account the end-state of their movement when first planning their movements. Rather, they employ alternative planning strategies (Wilmut and Byrne, 2014a,b). In line with this argumentation, previous studies that used an octagon task in which children had to rotate a knob, found that children used varying strategies including optimization of start-state comfort, reduction of initial rotation, and repetition of the previous movement (Wilmut and Byrne, 2014b). Additional research showed that children with DCD were more likely to use a strategy with minimal initial rotation of the hand and arm if compared with TD children (van Swieten et al., 2010; Wilmut and Byrne, 2014a). It was already mentioned in these studies that this pattern of results does not necessarily imply a lack of motor planning ability in children with DCD, but rather that these children may plan their grasps commensurate with their motor ability (van Swieten et al., 2010; Wilmut and Byrne, 2014a). Our results extend this argument by showing that both, children with DCD and TD children, are more likely to plan for ESC if task demands are more complex, as in the case of the present hammer-task.

We did not find statistically significant differences between children with DCD and TD children. At the individual level, however, the pattern of results was in line with our hypothesis that the difference in performance between both groups would be smaller on the hammer-task than on the sword-task. On the sword-task, six TD children ended more than half of the trials in a comfortable posture, while this was the case for only one child with DCD. On the hammer-task, seven TD children completed all trials in a comfortable posture, but two children ended less than half of the trials in a comfortable posture. For the children with DCD, two children completed all trials in a comfortable posture and there were three children that ended less than half of the trials in a comfortable posture. The lack of statistically significant differences between both groups is likely due to the small sample size of the present study which results in low power. This is supported by the results of the larger study including 26 children with DCD and 26 matched controls, where we did find that TD children demonstrated a higher percentage of ESC on the sword-task than children with DCD (Krajenbrink et al., in prep). The findings in the present study warrant fully powered follow-up research to test whether the pattern of results can be replicated in larger groups of children with DCD and TD children in order to draw strong conclusions. In addition, the order of the tasks should be counter-balanced to more systematically assess the probable confounding effects of practice and attention.

In sum, our small scale pilot study is the first to clearly show that both, children with DCD and TD children, are more likely to plan for ESC when high end-precision demands are combined with speed and force demands, as is the case in the hammer-task. These additional task demands were considered to increase the benefits to use an uncomfortable start-posture in order to end the movement in a comfortable posture. Indeed, our results indicate that while children with and without DCD plan their movements less consistently than adults on many previously used motor planning tasks, they are able to take into account the end-state of their movement and plan for ESC if demanded by task constraints.

## Data Availability Statement

The raw data supporting the conclusions of this article will be made available by the authors, without undue reservation.

## Ethics Statement

The studies involving human participants were reviewed and approved by the Ethics Committee Social Science of the Radboud University. Written informed consent to participate in this study was provided by the participants’ legal guardian/next of kin.

## Author Contributions

All authors have contributed to the work in a meaningful way. HK, JL, and BS designed the experiment. HK conducted the experiment, analyzed the data, and wrote a first draft of the manuscript. JL and BS critically reviewed the manuscript. All authors agree with publication of the final version of the manuscript.

### Conflict of Interest

The authors declare that the research was conducted in the absence of any commercial or financial relationships that could be construed as a potential conflict of interest.
